# Acoustic Feedback in Gait Rehabilitation—Pre-Post Effects in Patients With Unilateral Hip Arthroplasty

**DOI:** 10.3389/fspor.2021.654546

**Published:** 2021-05-07

**Authors:** Julia Reh, Gerd Schmitz, Tong-Hun Hwang, Alfred O. Effenberg

**Affiliations:** Institute of Sports Science, Leibniz University Hannover, Hannover, Germany

**Keywords:** gait sonification, hip arthroplasty, acoustic feedback, gait rehabilitation, range of motion, training intervention

## Abstract

It is known that patients after unilateral hip arthroplasty still suffer from a deficient gait pattern compared to healthy individuals one year after surgery. Through the method of gait sonification, it may be possible to achieve a more efficient training and a more physiological gait pattern. Increased loads on the musculoskeletal system could thus be reduced and rehabilitation times shortened. In a previous investigation with this patient group, we found immediate gait pattern changes during training with dual mode acoustic feedback [real-time feedback (RTF) and instructive model sequences (IMS)]. To determine whether an effect persists without the immediate use of acoustic feedback, we analyze data from four times of testing. Following unilateral hip arthroplasty 22 patients participated in an intervention of ten gait training sessions of 20 min each. During gait training the sonification group (SG) (*n* = 11) received an acoustic feedback consisting of RTF and IMS compared to a control group (CG) (*n* = 11). Pre-test, intermediate test, post-test, and re-test were conducted using an inertial sensor-based motion analysis system. We found significant effects (α = 0.05) regarding step length and range of motion (RoM) of the hip joint. Step length of the affected leg increased in the SG from intermediate test to post-test but decreased in the CG [intermediate test: (SG) 0.63 m ± 0.12 m, (CG) 0.63 m ± 0.09 m; post-test: (SG) 0.66 m ± 0.11 m, (CG) 0.60 m ± 0.09 m]. However, from the post-test to the re-test a reverse development was observed [re-test: (SG) 0.63 m ± 0.10 m, (CG) 0.65 m ± 0.09 m]. Also, from post-test to re-test a decrease in the RoM of the unaffected hip for the SG but an increase for the CG could be observed [post-test: (SG) 44.10° ± 7.86°, (CG) 37.05° ± 7.21°; re-test: (SG) 41.73° ± 7.38°, (CG) 40.85° ± 9.28°]. Regarding further parameters, significant interactions in step duration as well as increases in stride length, gait speed, cadence, and a decrease in ground contact time from pre-test to re-test were observed for both groups.

**Clinical Trial Registration:** https://www.drks.de/drks_web/, identifier DRKS00022570.

## Introduction

After unilateral hip arthroplasty, many patients still suffer from an unphysiological gait pattern even after several years (Queen et al., 2011; Kolk et al., 2014; Leijendekkers et al., 2018; Cezarino et al., 2019). This not only increases the strain on previously unaffected joints and other physiological structures, but also the risk of falling (Ninomiya et al., 2018). In order to return to a healthy gait, regular training is required beyond the usual rehabilitation period of about 4–8 weeks duration. New technological developments that can be used independently by the patient could provide efficient support for training and recovery (Krishnan et al., 2016; Chamorro-Moriana et al., 2018; Escamilla-Nunez et al., 2020). In this context, new feedback technologies make use of the fact that the human nervous system continuously compares its own motion with incoming somatosensory information and adjusts accordingly. This is exploited by either amplifying or artificially generating relevant external stimuli so that a comparison between motor behavior and visual, tactile, kinaesthetic or auditory perception is enhanced. The described method is called augmented feedback, which is a generic term for a wide variety of procedures including verbal feedback, error feedback and real-time feedback (Ronsse et al., 2011; Gilgen-Ammann et al., 2018; Bigras et al., 2019). It can generally be assumed that augmented feedback can improve motor learning (Sigrist et al., 2013) as extended feedback for rehabilitation has already been investigated in various clinical and applied studies (Storberget et al., 2017; Kearney et al., 2019; Melero et al., 2019).

In addition to visual, kinaesthetic and tactile feedback, acoustic feedback systems have also gained increasing interest in recent years in research on gait rehabilitation. Since walking is a cyclical movement that is determined by a rhythmic, reciprocal heel strike, research in this area has mainly focused on rhythmic auditory stimulation. For example, Thaut et al. (1996) were able to show early on that rhythmic auditory stimulation positively influences spatio-temporal gait parameters of Parkinson's patients. Positive effects of auditory cues could also be found in stroke patients (Shin et al., 2015; Mainka et al., 2018) and patients with multiple sclerosis (Baram and Miller, 2007). Recent studies, such as those by Dotov et al. (2017), indicated that the gait of Parkinson's patients benefits more from rhythmic auditory cues with a physiological variability compared to isochronous cues and Bella et al. (2015) have found a positive effect of signals that adapt to the gait kinematics of Parkinson's patients. Furthermore, in a gait study with healthy participants, Wu et al. (2020) were able to show that a change in cadence is better achieved by adapting acoustic cues than by fixed cues. This study (Wu et al., 2020) is an indication that a more targeted use of acoustic feedback, made possible by new motion analysis and sound systems, might provide further benefits for gait rehabilitation.

The study presented here is based on another form of acoustic feedback called motion sonification (Effenberg, 1996). It allows to reflect movements by sound in real time and thus to provide direct sensorimotor feedback that goes beyond the usual perception (augmented feedback). Kinetic or kinematic data are measured and mapped to sound by a defined function. Thus, a movement causes an immediate change or onset of the related sound, which therefore is directly influenced and created by the user. In order to create a succinct sound pattern and to achieve a close mapping between motion and sound, various musical parameters are used. Previous studies that investigated movement sonification could show that it can improve motor learning and motion adaptation in sports and rehabilitation (Schmitz et al., 2013, 2014; Effenberg et al., 2016; Schaffert and Mattes, 2016; Schaffert et al., 2019).

To effectively use sonification for patients after hip arthroplasty we developed a new acoustic feedback approach, which is based on a combination of kinematic real-time feedback (RTF) and instructive model sequences (IMS). A consistent sound in accordance with the human gait pattern was developed and applied, based on kinematic data recorded by a portable inertial sensor system. RTF is based on selected kinematic parameters (ground contact of the feet and angular velocity of the knee joint), which are clearly mapped to a sound. This means that each ground contact and each knee extension of the patient triggers the onset, frequency, and amplitude of a defined sound with low latency. On the other hand, IMS present the same sound as used for RTF, but in a predefined manner. Consequently, IMS display acoustic information at a fixed tempo, which is comparable to cueing movements.

Though, as far as known, there have been only a small number of studies investigating the influence of gait sonification in orthopedic patients (Yang et al., 2012; Pietschmann et al., 2019). In addition, very different study designs related to general acoustic feedback in gait training are reported in the literature, raising the question of the extent to which habituation to acoustic feedback and intervention sequences and durations are critical for effective use. For example, there are some studies referring to the immediate influence of acoustic feedback on gait pattern (Baram and Miller, 2007; Dotov et al., 2017). Others, however, are designed as intervention studies and compare pre-test and post-test data after 2 weeks (Pietschmann et al., 2019), 3 weeks (Thaut et al., 1996) or 4 weeks (Bella et al., 2015; Shin et al., 2015; Mainka et al., 2018) of gait training of varying frequency (3–7 times per week). In order to provide more detailed information on the effectiveness of gait sonification beyond the direct use (as published previously in Reh et al., 2019), this study presents results on pre-, post- and retention effects of the intervention with regard to the gait pattern of patients after unilateral hip arthroplasty. Due to the unilateral restriction of the patients, the gait symmetry in particular will be considered in the analysis. Furthermore, the aim is to determine whether a possible effect on the gait pattern can still be observed 2 days after the end of the intervention (re-test).

## Materials and Methods

### Patients

Twenty-two patients after unilateral hip arthroplasty were randomly assigned to either a sonification group or a control group. The patient recruitment and the study intervention were conducted in cooperation with a local rehabilitation clinic (Rehabilitationsklinik Niedersachsen, Bad Nenndorf). Every patient was admitted to the same rehabilitation clinic and thus followed a similar rehabilitation program. A pre-selection of the patients was carried out by the initial medical examination of the clinic, so that patients who showed additional medical risks or had severe pain were not admitted to the study. The inclusion criteria were defined as unilateral hip arthroplasty between 1 and 8 weeks ago, hospital admission for rehabilitation in the clinic for at least 2 weeks, walking ability with walking aids, and an age between 35 and 75 years. Patients with further arthroplasties, severe overweight, pacemakers, neurological diseases or hearing impairment were not recruited for the study. The study was conducted in accordance with the guidelines stated in the Declaration of Helsinki and the regulations of the Ethical Committee of the Leibniz University Hannover (EV LUH 02/2016). A total of 22 patients participated in the measurements and the 10-day gait intervention over a period of 8 months. Each participant received a written and oral explanation of the course of study and gave his or her written consent to participate voluntarily. Patients were divided into a sonification group (*n* = 11) and a control group (*n* = 11) and were parallelized according to age, height, body mass, gender, and the results of two clinical tests (timed-up and go test and sit-to-stand test within 30 s). The basic characteristics of the groups are shown in Table 1 and the results of the clinical tests are given in Table 2.

**Table 1:** Basic characteristics of the sonification group (SG) and control group (CG).

	**SG (*n* = 11)**	**CG (*n* = 11)**
Days post-surgery	15.27 ± 10.75	13.18 ± 4.67
Age [years]	62.9 ± 11.6	61.9 ± 7.9
Height [cm]	174.7 ± 5.3	178.0 ± 7.4
Body mass [kg]	86.4 ± 11.7	86.6 ± 12.5
Gender	9 male/2 female	8 male/3 female

*Values are mean ± SD*.

**Table 2:** The results of the clinical tests (timed-up and go and sit-to-stand test) for the sonification group (SG) and the control group (CG).

**Clinical test/group**	**Pre-test**	**Interm. test**	**Post-test**	**Re-test**	***t***	t^*^g
**Timed-up and go** [*s*]						
SG	11.78 ± 2.78	10.37 ± 3.69	8.74 ± 2.38	8.54 ± 2.36	<0.001; 1–β: 0.94	0.238; 1–β: 0.99
CG	13.85 ± 5.93	10.64 ± 2.97	10.63 ± 4.51	9.18 ± 1.51		
**Sit-to-stand test** [*repetitions per 30s*]						
SG	12.3 ± 4.5	14.5 ± 5.5	16.2 ± 6.5	17.8 ± 7.5	<0.001; 1–β: 0.99	0.948; 1–β: 0.16
CG	9.7 ± 5.3	12.4 ± 4.7	13.8 ± 5.2	15.4 ± 4.1		

*The p-values of the statistical analysis (ANOVA) are given in the right table section*.

The factors time (t) and time^*^group (t^*g^) were analyzed. Values are mean ± SD.

### Intervention

Both the sonification and control groups participated in 10 gait training sessions (TS) of 20 min each during a two-week intervention. Only the sonification group received dual-mode acoustic feedback during training. A pretest was performed before the intervention started, including a timed-up and go test and a sit-to-stand test (Table 2). In addition, a kinematic gait analysis was performed using MVN Awinda (XSens Technologies B.V., Enschede, Netherlands). Hearing ability of the SG was measured using HTTS hearing test software (Version 2.10, SAX GmbH, Berlin, Germany) and cadence was determined during 1 min of walking. At baseline, no significant differences between SG and CG neither for group characteristics nor for clinical tests could be found (days post-surgery *p* = 0.561, age *p* = 0.815, height *p* = 0.247, body mass *p* = 0.972, BMI *p* = 0.541, sit-to-stand test: *p* = 0.237, timed-up and go test: 0.262).

**Figure 1:**
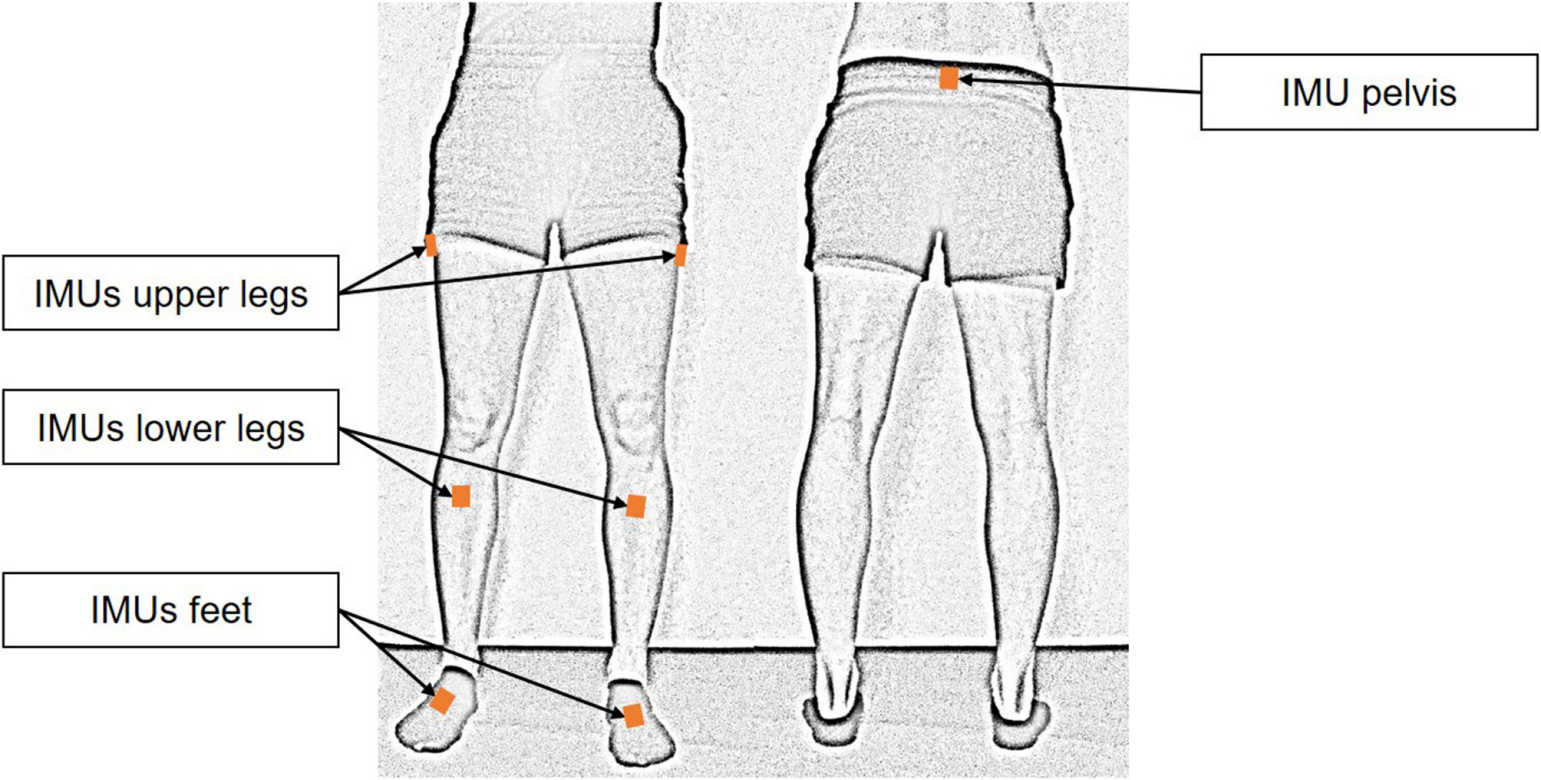
Positioning of the inertial measurement units. Seven sensors were fixed to the patient's body with Velcro straps according to the specifications of the MVN Awinda system.

In each gait training session, patients walked for 20 min in the rehabilitation clinic's 12 m × 15 m gym. During gait training, a laptop was placed in the gym to show the patients the temporal progress of the training. To enable sonification and motion analysis during training, patients in the sonification group and patients in the control group wore the wireless inertial sensor system MVN Awinda with inertial measurement units (IMUs) at the default specified by the system [sacrum (1 IMU), lateral side of femoral shafts (2 IMUs), medial surface of tibias (2 IMUs) and both feet (tarus) (2 IMUs)] (Figure 1). After each training session, patients in both groups received feedback on the distance covered, the steps taken and the gait speed.

**Figure 2:**
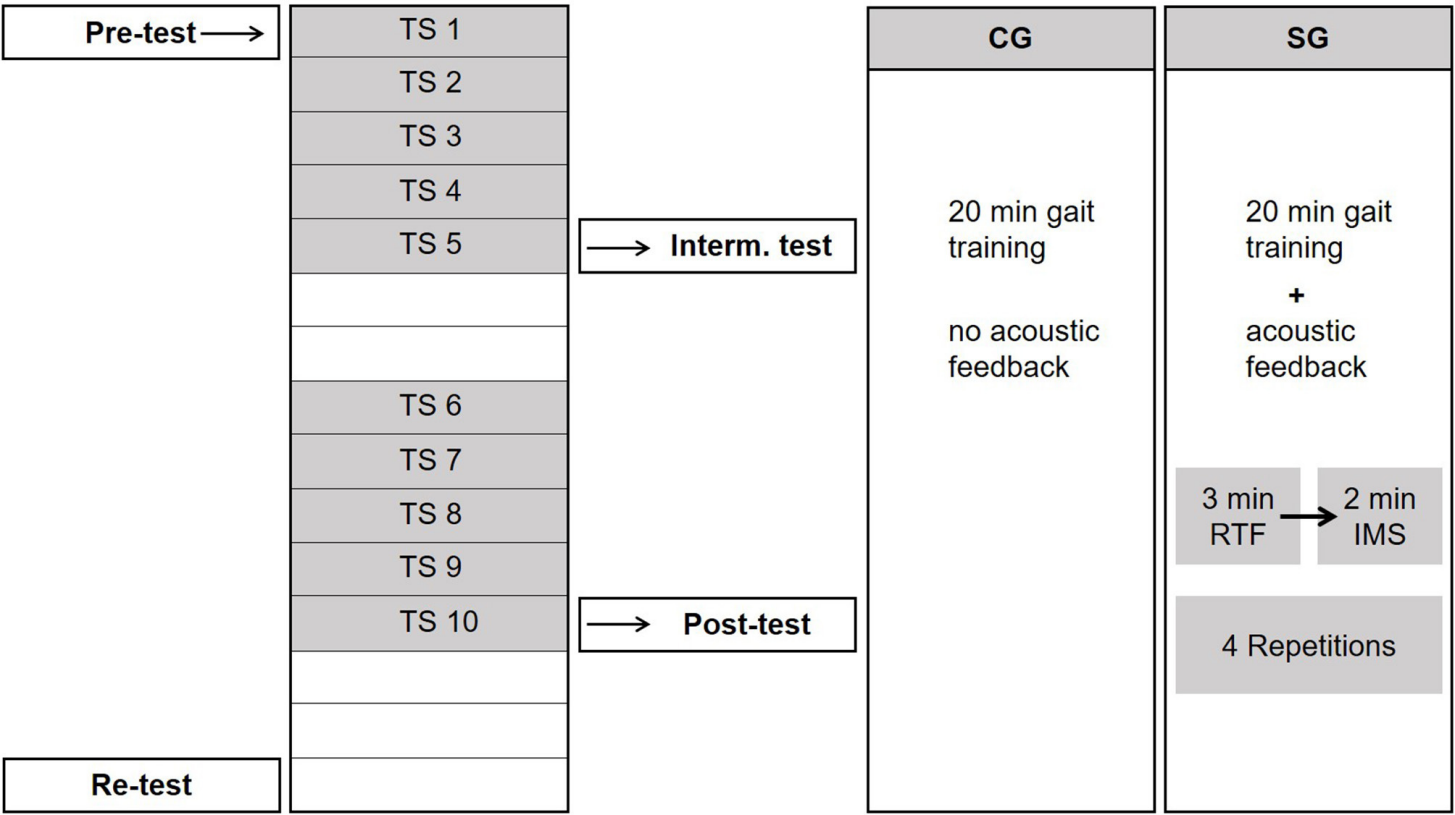
Process of intervention with 10 training sessions (TS) spread over 12 days. The control group (CG) did not receive any acoustic feedback, while the sonification group (SG) received real-time feedback (RTF) alternating with instructive model sequences (IMS).

Kinematic gait data was recorded at four measurement dates. The first measurement (pre-test) took place directly before the first training session. Subsequently, a second measurement took place after the fifth training session (intermediate test), followed by a third measurement after the tenth training session (post-test). On the second day after the end of the intervention the fourth measurement (re-test) was conducted (Figure 2). The wireless motion analysis system MVN Awinda (Xsens Technologies B.V., Enschede, Netherlands) and the software MVN Studio BIOMECH (Version 4.1., Xsens Technologies B.V., Enschede, Netherlands) were used to record kinematic data of the lower body. The patients walked a straight distance of 10 m with walking aids at a self-selected speed six to eight times at each measurement date. In addition, a timed-up and go test and a sit-to-stand test were performed.

### Dual Mode Acoustic Feedback

Due to the sequences of the acoustic feedback the gait training sessions of the sonification group were divided into 4-min blocks: Each block consisted of 3 min RTF and 2 min IMS. RTF, providing a low-latency feedback (<100 ms) of the patients' real gait pattern, was realized by direct data streaming out of the MVN Studio BIOMECH software to Spyder (Version 2.3.5.2., The Scientific Python Development Environment, Spyder Developer Community). In Spyder, an algorithm for detecting touch-down and toe-off of the feet as well as knee extension phase of the right and left leg during gait was applied. The generated kinematic events and periods (ground contact time and knee extension) were synthesized by an implemented Csound (Csound 6, LGPL) module resulting in a succinct sound pattern: The ground contact of the foot can be described in analogy of sound emerging when walking through heavy snow. Knee extension was acoustically represented as a sequence of xylophone strokes, usually a row of 5–7 quickly ascending tones per extension for healthy gait. Consequently, a whole gait cycle resulted in two successive snow compression sounds, each complemented by the xylophone of the contralateral knee extension. To enable a clear mapping between the sound and the according side of the body, the sound of the left leg (knee extension and ground contact of the foot) was four half tones (major third) lower than the sound of the right leg. Further, only the right speaker of the headphone gave the sound of the right leg while the left speaker gave the sound of the left leg.

The same sound pattern was used to generate IMS. Consequently, during IMS mode the patients heard synthesized “walking through snow” sounds and “xylophone strokes” in a fixed tempo, which was chosen based on body height and cadence. More precisely, IMS sounds were pre-recorded based on kinematic data sets to instruct a symmetric gait pattern. RTF and IMS were displayed successively and cumulated in 5 min blocks as it was intended to use enhanced sensorimotor representations formed during RTF for motor planning and execution during IMS. Therefore, exactly the same sound pattern was applied for RTF as well as for IMS. The kinematic data sets to produce a symmetric gait pattern sound were calculated as described in Reh et al. (2019). To ensure that IMS acoustically provide a symmetric gait pattern, kinematic data of the right and left leg were shifted by half a gait cycle. The datasets were synthesized and recorded to complete the new gait sonification method.

### Data Acquisition and Data Processing

The wireless motion analysis system MVN Awinda (Xsens Technologies B.V., Enschede, Netherlands) and the software MVN Studio BIOMECH (Version 4.1., Xsens Technologies B.V., Enschede, Netherlands) were used to record kinematic data of the lower body. This is an IMU based motion analysis system that can be used outside of laboratory conditions. A study by Zhang et al. (2013) indicates a high correlation (coefficient of multiple correlation > 0.96) for joint movements of the lower body in flexion-extension compared to a camera-based system. The gait events touch down (TD) and toe off (TO) were defined based on the acceleration data of the foot sensors. A self-developed MATLAB algorithm (R2016a, The MathWork Inc., Natick, MA, USA) was used for the standard detection of the gait events. In this algorithm a TD is defined as the minimum vertical foot acceleration provided that the corresponding foot is in front of the other foot. A TO is defined as the maximum vertical foot acceleration provided that the foot is behind the other foot. Due to this definition, steps are only included if one foot has passed the other. In this respect, the new algorithm differs from that used in the previous article (Reh et al., 2019), which limited search fields for peak detection solely by the position and speed of the respective foot sensor. A comparison of the two algorithms with optically evaluated TD (*n* = 1998) (and TO) events showed a significant higher accuracy of the new algorithm with a root mean square error (RMSE) of 0.75 ms for the old algorithm and 0.03 ms for the new algorithm. To assess the effect of the gait sonification method on the gait pattern, the parameters range of motion (RoM) of both hip joints, step length, stride length, step duration, stride duration, gait speed, cadence, and ground contact time were calculated and used for statistical analysis.

We defined one stride as the range between the TD of one foot to the following TD of the same foot. The hip angle of each stride was normalized to one hundred frames. One step was defined as the range from the TD of one foot to the following TD of the other foot. The gait speed is the average speed that the patients reached when walking the 10 m distance and the cadence is the step frequency as number of steps per minute.

### Statistical Analysis

The results of the parameters are presented as mean values and standard deviations (mean ± SD). A three-factor mixed ANCOVA was applied to the parameters step length, step duration, RoM of the hip and ground contact time considering the factors time (pre-test, intermediate test, post-test, re-test), side (affected leg, unaffected leg), and group (SG, CG) as well as days post-surgery as covariate. A two-factor mixed ANCOVA was applied to stride length, stride duration, gait speed, and cadence considering the factors time (pre-test, intermediate test, post-test, re-test) and group (SG, CG).

All data were checked by a Shapiro Wilk test for the condition of normal distribution. Data distribution normality was not fully met for step length and ground contact time. Therefore, the relevant data were transformed inversely for statistical analysis. The assumption of normal distribution was accepted for all other parameters, so they were not transformed. Levene's test indicated that the assumption for homogeneity of the variances was accepted for all parameters (*p* > 0.05). The analyses were performed using the SPSS version 26 (Chicago, IL) and level of significance was set at α = 0.05. If a significant interaction effect was observed using ANCOVA, it was then investigated to which differences in the data this effect can be attributed. To detect within-persons effects, *post-hoc* tests with sequential Bonferroni correction were performed in MATLAB. In addition, to specify interaction effects between the two groups, ANOVAS were performed over two measurements each (pre-test/intermediate test, intermediate test/post-test, post-test/re-test), which were also corrected using sequential Bonferroni correction.

## Results

For the results of the clinical tests (sit-to-stand and timed-up and go) which are given in Table 2, a significant time effect could be found: a decrease in the time required for the timed-up and go test (*p* = 0.033) and an increase in the sit-to-stand test (*p* = 0.41) became obvious. No time × group interaction (timed-up and go *p* = 0.378, sit-to-stand test *p* = 0.887) was observed.

### Spatial Gait Parameters

The results of the spatial gait parameters are shown in Table 3. For stride length no significant effects could be observed. For step length there were no significant main effects of time and group, but a significant side effect [*F*(3, 54) = 5.573, *p* = 0.030, *f* = 0.243] was found. Additionally, an interaction effect of time × side × group [*F*(3, 54) = 3.106, *p* = 0.034, *f* = 0.149] could be observed. This interaction confirms that step length developed differently between groups across the four measurements.

**Table 3:** Spatial gait parameters at the four test dates for the affected and unaffected leg of the sonification group (SG) and the control group (CG).

	**Pre-test**		**Interm. test**		**Post-test**		**Re-test**		**t**	**s**	t^*^(s)^*^g	t^*^(s)^*^g^*^d
	**affected**	**unaffected**	**affected**	**unaffected**	**affected**	**unaffected**	**affected**	**unaffected**	***p***	***p***	***p***	
**RoM hip**												
SG [°]	22.91 ± 6.48	39.55 ± 7.62	23.05 ± 6.65	43.94 ± 6.48	27.04 ± 5.91	44.10 ± 7.86	26.20 ± 5.66	41.73 ± 7.38	0.509;	0.013;	0.209;	0.039;
CG [°]	22.14 ± 7.31	35.79 ± 7.81	27.28 ± 5.97	38.27 ± 7.50	26.70 ± 7.08	37.05 ± 7.21	27.73 ± 5.06	40.85 ± 9.28	1–β: 0.21	1–β: 0.82	1–β: 0.99	1–β: 0.99
**Step length**												
SG [m^−1^]	1.83 ± 0.03	1.73 ± 0.23	1.65 ± 0.32	1.65 ± 0.22	1.56 ± 0.28	1.55 ± 0.27	1.64 ± 0.30	1.47 ± 0.26	0.179;	0.03;	0.034;	0.201;
CG [m^−1^]	1.89 ± 0.19	1.70 ± 0.20	1.61 ± 0.22	1.69 ± 0.23	1.70 ± 0.26	1.56 ± 0.13	1.57 ± 0.22	1.60 ± 0.27	1–β: 0.18	1–β: 0.56	1–β: 0.99	1–β: 0.99
**Stride length**												
SG [m]	1.15 ± 0.17	1.15 ± 0.17	1.25 ± 0.19	1.25 ± 0.19	1.32 ± 0.21	1.32 ± 0.21	1.33 ± 0.21	1.33 ± 0.21	0.204;	-	0.699;	0.759;
CG [m]	1.13 ± 0.11	1.13 ± 0.11	1.23 ± 0.13	1.23 ± 0.13	1.25 ± 0.13	1.25 ± 0.13	1.29 ± 0.16	1.29 ± 0.16	1–β: 0.17		1–β: 0.94	1–β: 0.87

*The values are mean ± SD. The p-values of the statistical analysis (ANCOVA) are given in the right table section*.

The factors time (t), side (s) the interaction time^*^side^*^group (t^*^s^*^g), and time^*^side^*^group^*^days post-surgery (t^*^s^*^g^*^d) were analyzed. The level of significance was set at α = 0.05.

For the control group, *post-hoc* tests revealed significantly increased step length of the affected leg from pre-test to intermediate test (*p* < 0.001) and pre-test to re-test (*p* < 0.001). The sonification group showed significantly increased step length of the affected leg from pre-test to post-test (*p* < 0.001) and of the unaffected leg from pre-test to re-test (*p* = 0.001).

A different progress between groups became evident for the affected leg from intermediate test to post-test [*F*(1, 20) = 9.514, *p* = 0.018, *f* = 0.69] with an increase in the sonification group and a decrease in the control group as well as from post-test to re-test [*F*(1, 20) = 21.732, *p* < 0.001, *f* = 1.04] with a decrease in the sonification and an increase in the control group.

### Temporal Gait Parameters

The temporal gait parameters are given in Table 4. For gait speed, cadence, and ground contact time significant time effects could be revealed across groups. No significant effects were found for stride duration. For step duration significant interactions of time × side × group [*F*(3, 54) = 3.532, *p* = 0.021, *f* = 0.166] and time × side × group × days-post-surgery [*F*(3, 54) = 3.47, *p* = 0.025, *f* = 0.164] were found.

**Table 4:** Temporal gait parameters at the four test dates for the affected and unaffected leg of the sonification group (SG) and the control group (CG).

	**Pretest**		**Interm. test**		**Post-test**		**Re-test**		**t**	**s**	t^*^(s)^*^g	t^*^(s)^*^g^*^d
	**Affected**	**Unaffected**	**Affected**	**Unaffected**	**Affected**	**Unaffected**	**Affected**	**Unaffected**	***p***	***p***	***p***	
**Step duration**												
SG [ms]	640 ± 96	631 ± 78	577 ± 86	560 ± 75	560 ± 84	544 ± 75	538 ± 58	535 ± 63	0.067 1–β: 0.25	0.708; 1–β: 0.07	0.021; 1–β: 0.99	0.025; 1–β: 0.99
CG [ms]	653 ± 104	651 ± 90	602 ± 101	582 ± 61	584 ± 73	570 ± 60	566 ± 62	548 ± 52				
**Stride duration**												
SG [ms]	1270 ± 171	1268 ± 171	1135 ± 159	1136 ± 159	1103 ± 157	1103 ± 155	1072 ± 120	1072 ± 119	0.075; 1–β: 0.24	0.708; 1–β: 0.07	0.375; 1–β: 0.99	0.289; 1–β: 0.99
CG [ms]	1305 ± 194	1302 ± 189	1182 ± 157	1181 ± 158	1153 ± 126	1153 ± 127	1115 ± 109	1114 ± 109				
**Ground contact**												
**time**												
SG [ms^−1^]	0.0013 ± 0.0002	0.0013 ± 0.0002	0.0015 ± 0.0002	0.0014 ± 0.0002	0.0015 ± 0.0002	0.0015 ± 0.0002	0.0016 ± 0.0002	0.0015 ± 0.0002	0.010; 1–β: 0.57	0.932; 1–β: 0.05	0.917; 1–β: 0.28	0.904; 1–β: 0.31
CG [ms^−1^]	0.0012 ± 0.0002	0.0013 ± 0.0002	0.0014 ± 0.0002	0.0014 ± 0.0002	0.0014 ± 0.0002	0.0014 ± 0.0002	0.0015 ± 0.0002	0.0015 ± 0.0002				
**Gait speed**												
SG [m^*^s^−1^]	0.91 ± 0.24		1.11 ± 0.28		1.20 ± 0.31		1.23 ± 0.28		0.010 1–β: 0.42	-	0.911; 1–β: 0.32	0.969; 1–β: 0.16
CG [m^*^s^−1^]	0.84 ± 0.16		1.02 ± 0.21		1.06 ± 0.20		1.13 ± 0.22					
**Cadence**												
SG [steps^*^min.^−1^]	88.95 ± 12.43		101.23 ± 12.98		103.21 ± 11.82		104.24 ± 9.09		0.003; 1–β: 0.50	-	0.781; 1–β: 0.50	0.608; 1–β: 0.86
CG [steps^*^min.^−1^]	86.47 ± 11.91		94.74 ± 12.02		96.25 ± 9.79		100.16 ± 9.61					

*The values are mean ± SD. The p-values of the statistical analysis (ANCOVA) are given in the right table section*.

The factors time (t), side (s) the interaction time^*^side^*^group (t^*^s^*^g), and time^*^side^*^group^*^days post-surgery (t^*^s^*^g^*^d) were analyzed. The level of significance was set at α = 0.05.

The interaction effects observed for step duration could be explained by *post-hoc* tests as follows: For the sonification group, *post hoc* tests revealed significant decreased step durations from pre-test to post-test (*p* = 0.037) and from pre-test to re-test (*p* < 0.001) for the affected leg. The same development could be observed for the unaffected leg with decreased step durations from pre-test to post-test (*p* = 0.015) and pre-test to re-test (*p* = 0.003).

For the control group, *post hoc* tests revealed significant decreased step durations from pre-test to re-test (*p* = 0.016) but not from pre-test to post-test for the affected leg. Though, for the unaffected leg again a decreased step duration from pre-test to post-test (*p* = 0.029) and from pre-test to re-test (*p* = 0.001) could be found.

### RoM of Hip Joint Angle

**Figure 3:**
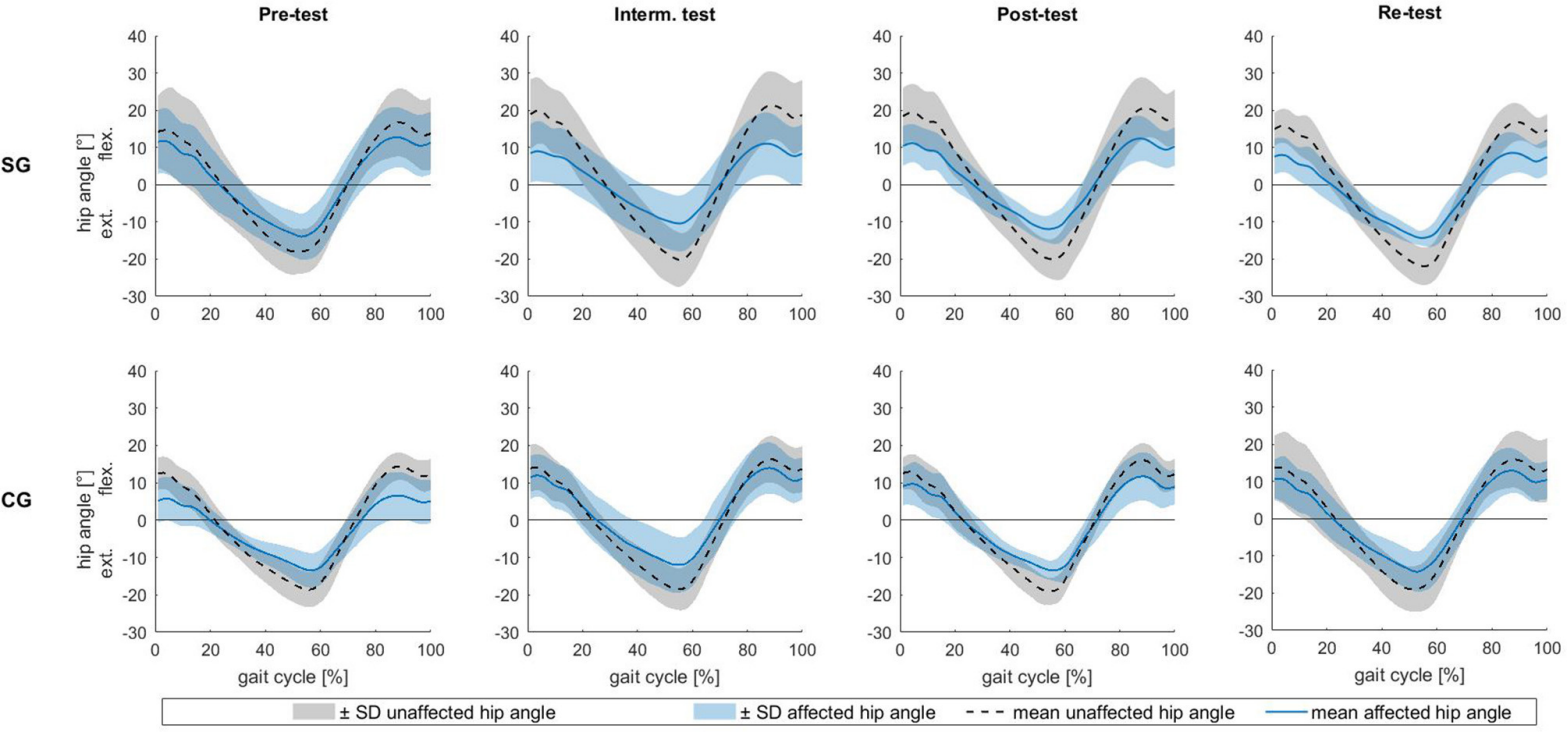
Hip joint angle over a gait cycle normalized to 100%. The lines (black: unaffected leg, blue: affected leg) are mean values. The shaded area above the line is the mean + 1 SD, the shaded area below the line is the mean −1 SD. The upper row shows the results of the sonification group (SG), the bottom row shows the results of the control group (CG) for the four test dates.

The measured values of the range of motion are given in Table 3 and mean hip joint angles of the affected and unaffected leg standardized to one stride are shown in Figure 3.

The results of the RoM of the hip joint angle revealed a significant side effect [*F*(3, 54) = 7.541, *p* = 0.013, *f* = 0.647]. Additionally, a significant side × time × group × days-post-surgery interaction [*F*(3, 54) = 2.996, *p* = 0.039, *f* = 0.409] was found. *Post hoc* tests showed a significant increase of the RoM of the affected hip joint angle of the control group from pre-test to intermediate test (*p* = 0.018) and from pre-test to re-test (*p* = 0.018). For the sonification group, *post hoc* tests revealed no significant within-person effects.

Furthermore, the RoM of the unaffected leg developed significantly differently between groups from post-test to re-test [*F*(1, 20) = 12.315, *p* = 0.007, *f* = 0.89]. In the sonification group, the RoM decreased from post- to re-test, but in the control group it increased.

## Discussion

**Figure 4:**
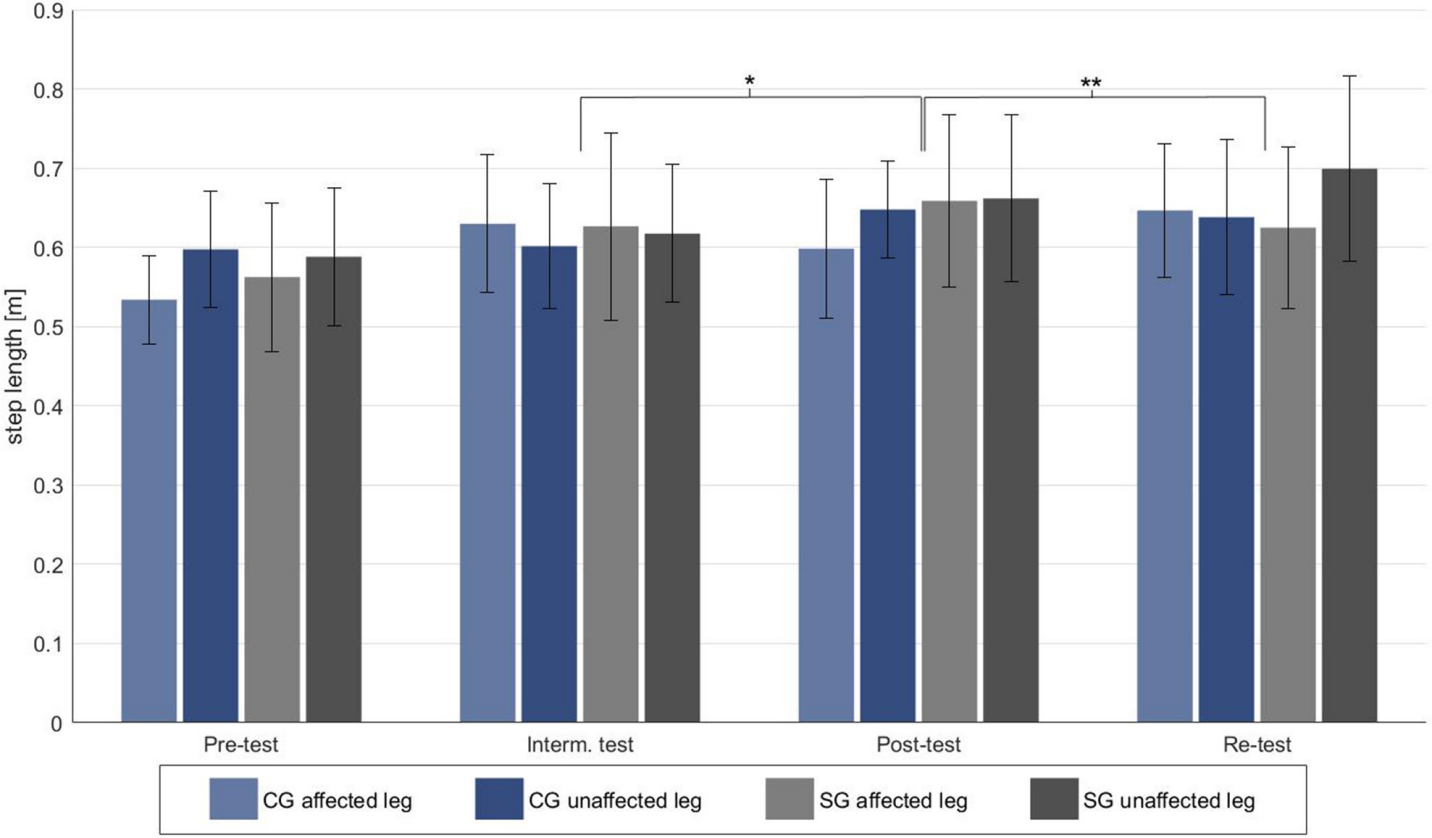
Step length of the affected and unaffected leg for the four test dates. Significant differences are marked with a *p < 0.05 and with a **p < 0.001.

Our results indicate an effect of the sonification method on the gait pattern of patients after unilateral hip arthroplasty. In particular, a significant effect of sonification on step length and RoM of the hip joint was found. An increase of step length of the affected leg in the sonification group from intermediate test to post-test, but a decrease in the control group could be observed (Figure 4). However, the re-test subsequently showed a reversal again, with a decrease in the step length of the affected leg in the sonification group and an increase in the control group. Additionally, a decrease in the RoM of the unaffected hip joint was noted for the sonication group from post-test to re-test in contrast to an increase for the control group. This is particularly noticeable because the post-test measurement took place directly after gait training, but the re-test was not preceded by gait training. For this reason, at least a short-term effect of gait sonification on the gait pattern can be assumed. Though, it can also be concluded that the acoustic dual-mode feedback did not lead to a stable change in gait pattern after 2 weeks of intervention. The observed step length asymmetry of the sonification group in the re-test is mainly due to an increased step length of the unaffected leg. In contrast, the control group showed improved step symmetry in the re-test. It can thus be stated that the method, in the context in which it was applied in the present study, did not lead to a clearly and sustainably improved gait pattern of the patients. Currently, the use of the method in clinical rehabilitation does not seem to be recommendable. However, the data provide first indications that the method is effective, since different developments between the groups could be observed in a short period of time (5 days each from intermediate test to post-test and post-test to re-test), so that further research in this field seems reasonable.

A basic improvement of general gait parameters (gait speed, cadence, stride length) and gait symmetry was expected in both subgroups, as they were recovering from a surgical procedure on the hip joint and usual training measures took place in the rehabilitation clinic. In this regard, Bahl et al. (2018) demonstrated improvement in gait speed, stride length, step length, and hip RoM 6 weeks after surgery in patients following hip arthroplasty. Rapp et al. (2015) also found increasing improvement in gait speed and gait symmetry in 29 patients after total hip arthroplasty when measured at days 15, 21, and 27 after surgery. In our study, the improvement in both groups might be attributed to the additional gait training through the participation in the study.

However, it is also known that even 12 months after surgery, deficits in gait can be found compared to healthy individuals of the same age (Queen et al., 2014; Bahl et al., 2018) indicating that it is a great challenge for patients to relearn a symmetric and steady gait pattern. Usually gait rehabilitation after hip or knee arthroplasty is associated with a large effort of time and personnel (Ong et al., 2015; Sabeh et al., 2017). In addition, it must be considered that prevalence of arthrosis increases with age (Neogi and Zhang, 2013; Allen and Golightly, 2015) and thus often affects elderly patients who suffer from several co-morbidities such as cognitive impairments. In this regard, a gait rehabilitation system that does not require high attentional cost might be a powerful add-on to classical treatments. The present study can only provide a first insight into the use of gait sonification for patients after unilateral hip arthroplasty, and the results only provide information about a short period after surgery, though, they do provide clues to future targeted applications of this method.

Although previous studies on the effect of acoustic feedback have shown positive effects (Thaut et al., 1996; Aruin et al., 2003; Schmitz et al., 2014; Bella et al., 2015, 2018; Park et al., 2015; Shin et al., 2015; Young et al., 2016; Dotov et al., 2017; Ghai et al., 2018; Chang et al., 2019; van Criekinge et al., 2019), clear evidence in patients undergoing gait rehabilitation with orthopedic diseases or (neurologically) healthy individuals is still lacking at the current time. In this context, (Yang et al., 2012) investigated the influence of acoustic error feedback during walking on three unilateral transtibial amputated patients. The ground contact time was measured in real time by means of insoles containing force sensitive resistors. A signal tone was generated in case of an unequal relationship between right and left ground contact time. In this way it was possible to improve the gait symmetry in terms of trunk sway and ground contact time ratio from pre-test to post-test by training six times for 30 min. However, a comparison with a control group is missing, so that it cannot be excluded that regular gait training alone has a positive effect on gait symmetry even without additional feedback. In our study exactly this could be observed, since the control group also shows an improved symmetry of the ground contact time and of several other parameters at the end of the intervention.

Horsak et al. (2016) also used force insoles containing seven force sensors in a pilot study to investigate the effect of sonification of ground contact times on the gait of 12 healthy, younger persons. However, there was no intervention, but the immediate influence of five different sounds on the gait of the participants was investigated. The five sounds differed in terms of their synthesizing (bandpass filtered white noise, wavetable, fm-synthesis, sinusoidal oscillator, Karpus strong algorithm), the assignment of frequencies or pitches to the seven force sensors, and ultimately in their timbre. Under the gait sonification conditions, a reduced cadence and gait speed was observed compared to a condition without sonification. A similar result was obtained by Fischer et al. (2017) with a comparable methodology in 22 participants over 50 years of age. However, this effect may be due to the short duration of sonification, what might have led to that the participants were not yet fully accustomed to the acoustics and concentrated more on the sound during sonification conditions. The present study, on the other hand, could not show an influence on cadence and gait speed after a two-week intervention, which could be due to a longer period of habituation and the different population investigated.

One of the few studies investigating the influence of acoustic feedback on patients after unilateral hip arthroplasty was published by Pietschmann et al. (2019). Patients of a rehabilitation clinic with unilateral hip arthroplasty (*n* = 120) were included. The patients were divided into six different groups, each comprising 20 patients (visual feedback, virtual feedback, tactile feedback, acoustic feedback, no additional feedback, control group), and participated in a 14-day intervention. This consisted of six 30-min gait training sessions on a treadmill. A pre-test was performed at the beginning and a post-test at the end of the intervention. The gait parameters gait speed, stride length, ground contact time and RoM of hip and knee joints were analyzed. It became clear that only the gait speed of the groups changed significantly different, while otherwise improvements were observed for all groups. The acoustic feedback group showed better results in terms of hip RoM and stride length, but these effects were not significant. In comparison to the present study, it should be emphasized that Pietschmann et al. (2019) chose a quite similar approach. With regard to the results, it can be said that the present study also shows a strong basic effect of gait training which is not due to additional feedback. Indeed, patients after hip arthroplasty seem to be severely restricted in relearning a physiological gait pattern during rehabilitation. This is probably due to structural limitations and/or previous and current pain, which is why this population seems to benefit above all from functional gait training. Nevertheless, it should be noted that significant effects on the gait pattern due to acoustic feedback were clearly shown in the current study. This difference in results from the study by Pietschmann et al. (2019) might be caused by the free walking in a gym (not on a treadmill) and the use of walking aids in the present study, which may have led to greater freedom of movement beyond the automated gait pattern. Another reason could be the different mapping of the sound to the movement. Pietschmann et al. (2019) focused on the sounding of the hip joint angles. In contrast, we chose a more distal approach with the sonification of ground contact duration and knee extension. Here, the primary intention was not to adjust or improve the parameters selected for sonification, but to provide a clear and concise temporal feedback for the patient.

In a previous study (Reh et al., 2019), we investigated the immediate effect of gait training with dual-mode acoustic feedback in the same patient group. The results indicated that RTF leads to greater step variability compared to IMS. The previous study also showed an effect on stride length. This finding is supported by the current results. Although there was a significant improvement in the affected leg step length of the sonification group from pre- to post-test, it is noticeable that a deterioration in step length symmetry of the sonication group was observed from post- to re-test. This could indicate that at this point there was still a close dependence between the new gait pattern learned during the intervention and gait sonification. For this reason, the gait pattern could probably be maintained only for a short time after the end of the gait training, but not until the re-test two days after the end of the intervention. It would be interesting to investigate in a future study, whether an intervention period of at least 4 weeks would have resulted in a long-lasting change in gait pattern. This could provide important new insights since it can be assumed that walking tends to perpetuate previously learned motor representations, as walking involves a high number of repetitions of the same motor pattern over and over again.

In addition, it can be surmised that the effect on step length symmetry would have been more apparent if the intervention had been scheduled later in the rehabilitation process and the walking aids could have been omitted. This should be considered as a limitation of the study, as at the chosen intervention time, the use of walking aids, pain, and severe structural injuries may have affected motor relearning. In addition, it should be taken into account that the size of the sample examined in this study was small, although the power of the results is not limited due to the high power of the statistical analysis regarding important effects. Nevertheless, it might be necessary to repeat the study over a longer period of time with a larger selected sample size and considering comorbidities and duration after surgery. This would allow a more extensive evaluation of the effectiveness of the sonification method. In a further step, gait sonification could be used in conjunction with mental training to establish the desired individual and physiological gait pattern.

## Conclusion

Dual-mode acoustic feedback training shows first indications of an influence on gait pattern in patients after unilateral hip arthroplasty. A short-term improvement of the gait pattern in terms of optimized gait symmetry is supported by the present results, especially with regard to step length. Future studies could help to shed deep light on these indications and thus clarify how acoustic feedback can efficiently and permanently influence the gait pattern of patients after unilateral hip arthroplasty. In this regard, the time period of an intervention and the precise association of kinematics to sound should be considered more comprehensively. We consider it likely that the reorganization of a physiological gait pattern representation can be accelerated by complementary mental training with a model sound. This relationship should be investigated predominantly with further scientific studies, because physical training is usually closely limited for the patients and the establishment of a robust auditory model gait pattern via concomitant mental training should be helpful for a better assessment of one's own gait pattern to get back to a symmetrical physiological gait.

## Data Availability Statement

The raw data supporting the conclusions of this article will be made available by the authors, without undue reservation.

## Ethics Statement

The studies involving human participants were reviewed and approved by Zentrale Ethik-Kommission der Leibniz Universität Hannover/Ethical Committee of the Leibniz University Hannover. The patients/participants provided their written informed consent to participate in this study.

## Author Contributions

JR drafted the manuscript. AE, GS, and T-HH revised it critically for important intellectual content. AE developed the sonification. AE, GS, and JR developed the framework for gait sonification and participated in the development of the intervention. T-HH and JR contributed to the software application, sound synthesis and involved in data analysis. AE and GS conceived, and designed the study. JR conducted the intervention and measurements. All authors read and approved the version of the submitted manuscript.

## Conflict of Interest

The authors declare that the research was conducted in the absence of any commercial or financial relationships that could be construed as a potential conflict of interest.
